# Editorials

**Published:** 1890-05

**Authors:** 


### HOMŒOPATHIC PHYSICIAN,

A MONTHLY JOURNAL OF

HOMŒOPATHIC MATERIA MEDICA AND CLINICAL MEDICINE.

If our school ever give up the strict inductive method of Hahnemann, we are lost, and deserve only to be mentioned as a caricature in the history of medicine."—CONSTANTINE HERING.

Vol. X. MAY, 1890. No. 5.

### EDITORIALS.

MEDICINE AN EMPIRICAL ART.—" The practice of medicine is—even at its best—so empirical an art, the limitations of our best endeavors, when measured by our aspirations, are everywhere so narrow, that we should always be prepared to hail with joy any one department in which we discern *some* prospect of advancing with anything like scientific accuracy.

" Though pharmacology has made great strides in the past decade, though we are well acquainted with the physiological action, and even with the therapeutic value of drugs; yet, compared with the vast amount of labor already expended, how small, on the whole, is our success in combating disease by pharmaceutical agents."

We deem it superfluous to say to any Hahnemannian that the above comes from a regular (?) practitioner, and is found in an old-school journal. If one had not seen the title of the article, which has the above for a beginning, certainly, one would think here, and at last, we are to learn something new regarding the treatment of disease.

But let us not be too much amused, even though it was written in connection with " THE PHYSIOLOGICAL TREATMENT OF OBESITY," for we can thank the writer—or those who are too fat should—for again calling attention to the importance of diet in obesity.

It is true that, even though we lay down rules of diet for many who wish to be thinner, they will keep on being fat, until we recognize that obesity in most cases is disease, and must be treated homœopathically in order to be mastered.

However, we may gain from such articles as the above mentioned much that goes to prove the truth of our position, based on Hahnemann's indefatigable labors.

In the same article the writer, speaking of errors that have obtained regarding the cause of obesity, says: "It is but another example of the observation to which all of us must have been often led, namely, that the most obvious and plausible explanations of natural phenomena are almost invariably incorrect." Hahnemann had something to say on this subject, and by turning to our February number, p. 56, there will be found a few lines that fit here admirably. G. H. C.

---

Colleges, Journals, Pharmacies.—So-called homœopathic colleges, homœopathic journals, and homœopathic pharmacies there are in abundance, but the doubt is, where is their Homœopathy?

We are willing to offer a reward for a college where any one may learn Homœopathy pure and simple, without any feeble imitation of allopathic teaching. We are willing to increase this reward for the purpose of having pointed out to us any journal purporting to be homœopathic, with the exception of Dr. T. S. Hoyne's *Medical Visitor* and The Homœopathic Physician, which does not bear, either in its reading matter or advertisements, sufficient evidence to prove that it is prostituting the fair name of Homœopathy for the sake of a few dollars. As for pharmacies; are we safe in offering a larger reward to be shown one in which is not found all the makeshift appliances that are to be found in any ordinary drug-shop?

Indeed, in looking at the various journals bearing the title of homœopathic, hearing of the teachings in homœopathic (?) colleges, and seeing what is displayed in pharmacies, we can only conclude that a conspiracy has been entered into among these various tradesmen for the purpose of belittling true Homœopathy.

To know what is taught in the colleges one needs only to read the journals. And in reading the journals one readily sees how few there are who understand the true meaning of the term Homœopathy. And the advertisements! Compare them with an old-school journal. There is little or no difference. Illustration? A monthly bearing the name of *Homœopathy* a short time ago, in an editorial, strongly indorsed an excellent paper on "Antisepticism." This paper wholly condemned the use of any and all so-called antiseptics except cleanliness. With this, of course, we are at one. But, a few pages from this editorial was to be found an advertisement of "Listerine." And the editor of that journal is its proprietor, and has control of its advertising pages! The same journal is now engaged in disseminating, through its advertising pages, a sure cure for all cases of rupture. And yet this editor claims to be a consistent disciple of Hahnemann! Is this consistent? We should like to ask, Is it honest? Is it honest to condemn in editorial pages as hurtful things that receive indorsement at the same hands by being admitted to the advertising pages?

Honesty includes FAITHFULNESS.

Again, in another journal, whose editor is a professor—a teacher—in a homœopathic (?) college, we sometimes see editorials which have the true ring. Then we read that this editor—this teacher (?)—for hemorrhage after labor gave material doses of Ergot. Is this honesty? is this faithfulness? And so we may go from one journal to another, and with the exceptions noticed above, we find but little Homœopathy and much eclecticism. We find one or two pages, perhaps, given to homœopathic matter, and the rest filled with old-school commendation of various mixtures for pathological names, which any one having but an intimation of what Homœopathy can do can only turn from with disgust.

If these journals have any excuse for being—aside from the dollars their advertisements bring—we should be glad to have it shown us. Our vision does not extend so far as to enable us to see an excuse for them.

As for the pharmacies, we trust the time is not far distant when we shall have a truly homœopathic pharmacy; a place where only homœopathic appliances may be found; a place where we may be sure that our delicate remedies are prepared in a laboratory in which no mixtures of drugs are allowed, and where we may feel only that which is honest obtains.

We are not believers in boycotting, but we wish to ask if the time has not arrived for the followers of Hahnemann to take concerted measures to abate these dishonest practices which cling like barnacles to the good ship Homœopathy? G. H. C.

---

"Occasionally a regular [Hahnemann knew of the regulars] brother practitioner stumbles by a lucky hit upon a cure which astonishes half the world about him, and not less himself; but among the many medicines he employed he is by no means sure which did good. Not less frequently does the neck-or-nothing practitioner, without a degree, whom the world calls a quack, make as great and wonderful a cure. But neither he nor yet his wonderful brother practitioner with a diploma knows how to eliminate the evident and fruitful truth which the cure contains. Neither can separate and record the medicine which certainly was of use out of the mass of useless and obstructing ones they employed; neither precisely indicates the case in which it did good, and in which it will certainly benefit again. Neither knows how to abstract a truth which will hold good in all future time, an appropriate, certain, unfailing remedy for every such case that may occur hereafter. His experience in this case, remarkable though it seemed, will almost never be of service to him in any other. All that we learn is that a helpful system of medicine is possible; but from these and a hundred other cases it is quite manifest that as yet it has not attained the rank of a science, that even the way has yet to be discovered how such a science is to be learned and taught. As far as we are concerned it cannot be said to exist.

"Meanwhile, among these brilliant but rare cures there are many, vulgarly called *Pferdecuren* [horse cures], which, however great the noise they might make, are not of a character to be imitated, *salti mortali*, madly, desperate attempts by means of most powerful drugs in enormous doses, which brought the patient into the utmost imminent danger, in which life and death wrestled for the mastery, and in which a slight, unforeseen preponderance on the side of kind nature gave the fortunate turn to the case; the patient recovered and escaped from the very jaws of death.

"This cannot be the divine art, that like the mighty working of nature should effect the greatest deeds simply, mildly, and unobservably, by means of the smallest agencies."—Hahnemann, 1805.

---

SEQUELÆ.—We have always claimed—and we know that we can prove—that no sequelæ follow diseases treated by our law of therapeutics.

Should other affections follow an original malady—that is, a state which is new to the patient—by questioning we shall find that the patient had previously gone through a similar, or allied, condition, and that he had been treated in a way that caused the condition to disappear. But it was not cured—merely suppressed. And we may thus prove Hahnemann's observations on this point correct. We need not stop here. We can prove, by the practice of every Hahnemannian, that our patients are not liable to various affections which we see follow upon some physiological process, and which are said to be a part of this process, plus microbes. Principal among these is puerperal fever. Although our own obstetric practice has been limited, yet we have had a number of cases, and in not one have we had puerperal fever to treat. And we have never stepped aside from our law and resorted to makeshifts. We have always treated abnormal symptoms by the use of the potentized remedy alone.

The testimony of all Hahnemannians is to the same effect. We shall be pleased to have our readers who have ever had any difficulty arise after labor—that is, any affection which is said to be a concomitant of the puerperum—to give us their experience. In doing so, it will be of more value if we have given as complete a history of the case or cases, as is possible. And we shall be glad to have evidence which will strengthen the position we have taken on the subject. Thus we may learn just how far we are justified in maintaining that position. G. H. C.

---

COINCIDENCES.—In the March number of *The American Homœopathist* is a series of clever editorials by Dr. Frank Kraft, having the above title for their theme. In the third one, on page 86, is the report of a case of gall-stone colic which so strongly recalls an experience of our own that we are induced to relate it.

It was a case of *renal colic* in a woman. The patient was nominally under the care of our honored friend and benefactor, the late Dr. Lippe. But as he was unable to continue in attendance, owing to sudden illness, the care of the case fell to our lot in common with all the rest of his practice.

The patient had had numerous and terrible attacks for many years. She had always been treated by physicians of the old school of medicine, or else by eclectic homœopathists. Consequently she had been liberally dosed with Morphine, until her arms were studded with painful nodes and her reason very nearly dethroned.

At last, in complete despair, her friends telegraphed a statement of the case to Dr. Lippe. We were present in his office when the telegram came. It completely filled three telegraphic blanks. Dr. Lippe advised Lycopodium, *high.* The effect was so remarkable that the patient came to Philadelphia shortly afterward to continue his treatment.

It was then that she fell under our notice. For, after an unusually long interval of peace, one of the attacks came on. We were hastily summoned, and found the patient groaning loudly, and writhing about the bed like a serpent. Her sister was vigorously pressing her closed fist into the right inguinal region. But the patient constantly exclaimed: "Sister, press harder!" We learned that only this *hard pressure gave relief, whilst a slight touch aggravated instantly.*

Upon this indication we gave Nux-vomica$^{2c}$ in water. In ten minutes the patient was perfectly quiet. The relief was so remarkable and so quick that the patient accused us of giving Morphia. We had some difficulty in satisfying her suspicions.

"If you have given me Morphia," she added, "you may go out that door and not return, for I will never again take Morphia."

This was the last attack she experienced for some years. So here is another coincidence to offset the ambitious dispenser of lead quoted in the editorial. W. M. J.

